# Individual and country-level variables associated with the medicalization of birth: Céline Miani

**DOI:** 10.1093/eurpub/ckac129.191

**Published:** 2022-10-25

**Authors:** C Miani, L Wandschneider, S Batram-Zantvoort, B Covi, H Elden, I Hersoug Nedberg, Z Drglin, E Pumpere, R Costa, M Lazzerini

**Affiliations:** 1 School of Public Health, Bielefeld University, Bielefeld, Germany; 2 Ined, Aubervilliers, France; 3 WHO Collaborating Centre, Institute for Maternal and Child Health IRCCS, Trieste, Italy; 4 Institute of Health and Care Sciences, Sahlgrenska Academy, University of Gothenburg, Gothenburg, Sweden; 5 Department of Obstetrics and Gynecology, Sahlgrenska University Hospital, Gothenburg, Sweden; 6 Department of Community Medicine, UiT The Arctic University of Norway, Tromso, Norway; 7 National Institute of Public Health, Ljubljana, Slovenia; 8 Riga Maternity Hospital, Riga, Latvia; 9 Department of Obstetrics and Gynecology, Riga Stradins University, Riga, Latvia; 10 EPIUnit, University of Porto, Porto, Portugal

## Abstract

**Introduction:**

According to the World Health Organization, the medicalisation of birth tends “to undermine the woman's own capability to give birth and negatively impacts her childbirth experience”. The COVID-19 pandemic has disrupted maternity care, with potential increase in the medicalisation of birth and in occurrences of disrespectful maternity care. We aim to investigate potential associations between individual and country-level factors and medicalisation of birth in 15 European countries during the COVID-19 pandemic.

**Methods:**

We collected data through an online, anonymous survey for women who gave birth in 2020-2021. We ran multivariable, multi-level logistic regression models estimating associations between indicators of medicalisation (caesarean section (CS), instrumental vaginal birth (IVB), episiotomy, fundal pressure) and proxy variables related to care culture and contextual factors at the individual and country-level.

**Results:**

Among 27173 women, 24.4% had a CS, and 8.8% an IVB. Among women with IVB, 41.9% reported receiving fundal pressure. Among women with spontaneous vaginal births, 22.3% had an episiotomy. Less respectful care, as perceived by the women, was associated with higher levels of medicalisation. For example, women who reported having CS, IVB and episiotomy reported not feeling treated with dignity more frequently than women who didn't have those interventions (respectively: OR: 1.37; OR: 1.61; OR: 1.51; all: p < 0.001). Country-level variables contributed to explaining some of the variance between countries.

**Conclusions:**

We recommend a greater emphasis in health policies on the promotion of respectful and patient-centered care approaches to birth to enhance women's experiences of care, and the development of a European-level indicator to monitor the medicalisation of reproductive care.

**Speakers/Panellists:**

**Emanuelle Pessa Valente**

WHO Collaborating Centre, Institute for Maternal and Child Health IRCCS, Trieste, Italy

